# Pandemic lessons: protecting sexual and reproductive health during global health emergencies

**DOI:** 10.1186/s12978-026-02273-5

**Published:** 2026-02-16

**Authors:** Moazzam Ali, James Kiarie

**Affiliations:** Department of Sexual, Reproductive, Maternal, Child and Adolescent Health and Ageing and HRP, the UN’s Special Programme in Human Reproduction, World Health Organization, Geneva, Switzerland

**Keywords:** COVID-19 pandemic, Sexual and reproductive health, Health systems resilience, Service disruption, Telemedicine, Essential health services, Emergency preparedness, Health equity, Community health workers

The COVID-19 pandemic profoundly disrupted essential health systems worldwide, exposing structural vulnerabilities and testing their resilience. Among those most significantly impacted were Sexual and Reproductive Health (SRH) services, that are vital for protecting the health and rights of women, adolescents, and marginalized populations. This special supplement presents a synthesis of experiences of SRH service disruptions from nine countries Brazil, Burkina Faso, China, Ghana, Italy, Kenya, Pakistan, Thailand, and the United Kingdom [1]. Each country experience offers critical insights into the challenges, adaptations, and innovations that emerged in efforts to sustain SRH services during an unprecedented global emergency.

The studies document widespread disruptions in SRH services, including family planning, maternal and newborn care, access to contraception, safe abortion, and HIV/STI prevention and treatment,. Lockdowns, supply chain interruptions, workforce reallocation, and overwhelmed health facilities forced the postponement or suspension of routine care. Particularly vulnerable were underserved populations in rural areas and fragile health systems. Across settings, the initial designation of SRH services as “non-essential” contributed to reduced access, deepening inequities and reversing hard-won gains in reproductive health.

Despite diverse political, economic, and health system contexts, the nine countries shared common challenges: declining service availability, reduced mobility for clients, constrained provider capacity, and heightened barriers for vulnerable groups. Yet, what emerges equally strongly are shared patterns of adaptation. Countries pivoted to alternative service delivery models, with a commitment to protecting SRH access even under extreme constraints.

Telemedicine played a pivotal role, enabling remote consultations and follow-up care in countries as different as the UK and Thailand. Community-based service delivery was bolstered in Ghana and Pakistan through the mobilization of community health workers [2]. China and Thailand embedded SRH services within national COVID-19 response frameworks, ensuring continuity from the outset [3,4]. Regulatory flexibility, such as task-shifting, extended prescriptions, and support for self-care interventions allowed several countries, including Brazil and the UK, to maintain essential care despite facility-level disruptions [5].

Several key lessons emerge from these country experiences. First, Sexual and Reproductive Health (SRH) services must be designated as essential from the start of any crisis. Initial designation of SRH services as non essential not only cost lives but also deepened existing disparities and delayed integrating SRH into emergency preparedness and response plans from the outset. Second, deploying flexible and decentralized delivery models is critical. Systems that facilitated home-based care, mobile outreach, and digital health innovations were notably more effective in mitigating service disruptions. Third, local leadership and community engagement served as crucial pillars of resilience. Finally, policy adaptation enabled continuity of care. Rapid adjustments to guidelines and regulations allowed providers to respond effectively to emerging needs. Embedding such flexibility into routine health governance will be essential for ensuring future readiness.

The lessons from COVID-19 are clear: to prepare for future health emergencies, countries must institutionalize sexual and reproductive health (SRH) resilience as part of broader health system strengthening efforts. This involves investing in digital infrastructure to support telehealth and remote service delivery; strengthening community health systems and task-sharing models to increase reach and adaptability; ensuring that policy frameworks are flexible, rights-based, and inclusive of self-care and decentralized service delivery; and embedding SRH within emergency preparedness and universal health coverage (UHC) strategies.

This supplement is a timely and important contribution to the global health literature. It brings together a diversity of country experiences, reflecting a range of health system capacities, geographic contexts, and policy environments. The collective insights from these nine case studies offer not only a snapshot of challenges faced but also a compelling blueprint for future preparedness.

We commend the country teams whose work is reflected in these pages. Their documentation of lived experiences, local innovations, and policy responses will serve as a valuable reference point for researchers, implementers, and policymakers seeking to ensure that SRH services are protected and prioritized in future pandemics and public health emergencies [6].

As we look ahead, the COVID-19 pandemic must serve not only as a wake-up call but as a catalyst for building more equitable, inclusive, and resilient SRH systems. The future of SRH care in emergencies depends on the actions we take now anchored in equity, driven by evidence, and shaped by the voices of those most affected.

## Abbreviations

SRH: Sexual and Reproductive Health
STI: Sexually Transmitted Infection
HIV: Human Immunodeficiency Virus
UHC: Universal Health Coverage
